# Can AI-Based Video Analysis Help Evaluate the Performance of the Items in the Bayley Scales of Infant Development?

**DOI:** 10.3390/children12030276

**Published:** 2025-02-25

**Authors:** Dong Hyun Ye, Tae Won Kim, Su Min Kim, Ji Won Seo, Jongyoon Chang, June-Goo Lee, Eun Jae Ko

**Affiliations:** 1 Department of Rehabilitation Medicine, Asan Medical Center, University of Ulsan College of Medicine, Seoul 05505, Republic of Korea; ydh7342@amc.seoul.kr (D.H.Y.); d240133@amc.seoul.kr (J.C.); 2 Department of Biomedical Engineering, Asan Medical Center, University of Ulsan College of Medicine, Seoul 05505, Republic of Korea; taewon01964@amc.seoul.kr; 3 Department of Rehabilitation Medicine, Asan Medical Center, Seoul 05505, Republic of Korea; suemin5079@amc.seoul.kr (S.M.K.); jiwonseo@amc.seoul.kr (J.W.S.)

**Keywords:** developmental delay, machine learning, video analysis

## Abstract

**Aims:** To develop and evaluate a novel AI-based video analysis tool for the quantitative assessment of “Places Pegs in” and “Blue Board” tasks in the Bayley Scales of Infant Development (BSID-II). **Methods:** A prospective cohort study was conducted from February 2022 to December 2022, including children aged 12–42 months referred for suspected developmental delay. Participants were evaluated using the BSID-II, and their performances on the two tasks were video recorded and analyzed with the novel tool. Sensitivity and specificity were determined by comparing the tool’s results to standard BSID-II assessments by therapists. Data collected included total time, number of trials, successful trials, and time and spatial intervals for each trial. Children were classified into typically developing (TD) (MDI ≥ 85) and developmental delay (DD) (MDI < 85) groups based on their mental developmental index (MDI). **Results:** A total of 75 children participated in the study, and the mean values of MDI and PDI for the enrolled children were 88.9 ± 18.7 and 80.0 ± 16.7. The “Places Pegs in” had 86.5% sensitivity and 100% specificity; the “Blue Board” had 96.9% sensitivity and 89.5% specificity. Differences in cumulative successes over time were observed between age groups and TD and DD groups. The tool automatically calculated maximum successes at specific time points. **Interpretation:** The AI-based tool showed high predictive accuracy for BSID-II tasks in children aged 12–42 months, indicating potential utility for developmental assessments.

## 1. Introduction

Development refers to the process of acquiring skills and functional capabilities over time. Developmental delay (DD) is a term used to describe a significant delay or impairment in one or more areas of a child’s development, such as motor, cognitive, language, or social-emotional development. A significant delay is defined as a performance that falls two or more standard deviations below the average on standardized norm-referenced tests [1]. It can affect children of any age and is often caused by genetic or environmental factors [2,3]. Early identification of DD is crucial, as it allows timely intervention and the provision of support to maximize a child’s potential [4,5]. Without intervention, DD can have long-term effects on a child’s academic, social, and emotional well-being, resulting in developmental disorders. The literature on early brain development shows that optimal interventions during critical periods of child brain development become increasingly important [5]. In particular, since 85% of a child’s brain develops by the age of 5, the educational experiences during the first five years of life are an important foundation for future health and life success [5,6,7,8]. Therefore, early and accurate diagnosis of developmental delay in childhood is crucial.

The Bayley Scales of Infant Development (BSID) is a standardized assessment tool designed to measure the development of infants and toddlers from 1 to 42 months of age, which is the most widely used in the clinical and research setting. It assesses developmental domains, including cognitive, language, and motor skills [9,10,11,12]. The assessment is administered by trained professionals and involves direct observation of the child’s behavior during specific tasks. Results are reported as a standardized score that can be used to track developmental progress over time, identify areas of strength and weakness, and inform intervention and treatment planning. In these tests, the examiner directs the child to perform the prescribed test items, determines whether the child performs appropriately, and scores the performance to help diagnose DD. It is important to note that the test is limited in that the evaluation and interpretation must be carried out by highly trained experts with a comprehensive understanding of typical developmental processes. This is often challenging in areas lacking specialists or with limited access to healthcare [9,11]. Furthermore, in BSID, the evaluation process is time-consuming, and each item is presented with only two outcomes: credit or not credited [9].

The second edition of the Bayley Scales of Infant and Toddler Development (BSID-II) was a pioneering tool that was widely used for over a decade to assess cognitive and motor development in early childhood [9,11,12]. Its simple structure and strong psychometric properties made it a valuable resource in both clinical assessment and developmental research across diverse populations. The third edition (BSID-III) introduced the Social-Emotional and Adaptive Behavior scales, broadening the scope of developmental assessment. However, some studies have raised concerns that the developmental indices derived from the BSID-III tend to be higher than its predecessor (BSID-II), potentially underestimating DD [11,13,14]. The fourth edition (BSID-IV) incorporates digital tools to improve the efficiency of administration and scoring while incorporating the latest developmental theories and empirical findings [15]. Despite these advances, the transition to a digital platform can be challenging for examiners accustomed to traditional assessment methods.

The integration of artificial intelligence (AI) and automation into healthcare systems holds immense potential for bridging the global health disparity gap [16,17]. In low-resource settings, the shortage of trained healthcare professionals often limits access to quality care. AI technologies, including machine learning and remote monitoring tools, offer scalable solutions that can support accurate and timely assessments without the need for specialized expertise. Ultimately, this innovation could enhance healthcare access, improve early detection of DD, and reduce health inequities globally.

In recent years, there has been growing interest in the development of AI-based tools to evaluate motor development in infants and toddlers [18,19,20,21]. By using computer vision technology and machine learning algorithms, these assessments can automatically and quantitatively analyze and track an infant’s movements in real time. This approach reduces variability in individual results and enhances the accuracy of the assessment. Several AI-based tools have been reported in the literature. BabyNet is a deep learning-based network designed to evaluate infant reaching action (accuracy of 66%, positive predictive value of 0.57, and sensitivity of 0.72) [22]. The network leverages a lightweight architecture to achieve high accuracy in detecting infant reaching action while being computationally efficient. Another study proposed a detection-based approach to classify infants’ actions from multiple views (accuracy of 80%, positive predictive value of 0.79, sensitivity of 0.80) [23]. The proposed method can dynamically adjust to and accurately interpret different types and patterns of movements that infants may exhibit. It can provide accurate and reliable results across a variety of conditions and scenarios. A study that uses computer vision technology to assess infant neuromotor risk automatically has also been suggested (accuracy of 88%, sensitivity of 94%, specificity of 89%) [24]. This study proposes a deep learning framework for the analysis of infant movements and provides a risk score for each infant. Therefore, AI and computer vision technology have the potential to assess infant motor development. However, previous studies have been confined to video-based developmental assessment and are limited to the domains of spontaneous movement and gross motor skills. Accordingly, these studies have not been applied to behavioral tasks such as reaching for a bar or matching shapes, which are commonly used to assess child development, nor have they examined the association with BSID as it is currently used clinically. Therefore, further research is needed to develop more robust and practical tools for assessing real tasks performed by children other than the assessment of gross motor skills.

The primary objective of this study was to develop a novel AI-based video analysis to quantitatively analyze “Places Pegs in” and “Blue Board” items in BSID-II in children aged 12 to 42 months in a real clinical setting. The second objective was to compare the accuracy of this innovative novel tool with the results of the conventional BSID items and to explore the additional insights that can be gained using this novel tool. We expect this new tool to provide accurate developmental assessments, especially in areas where experts are not available.

## 2. Methods

### 2.1. Participants

**Figure 1:**
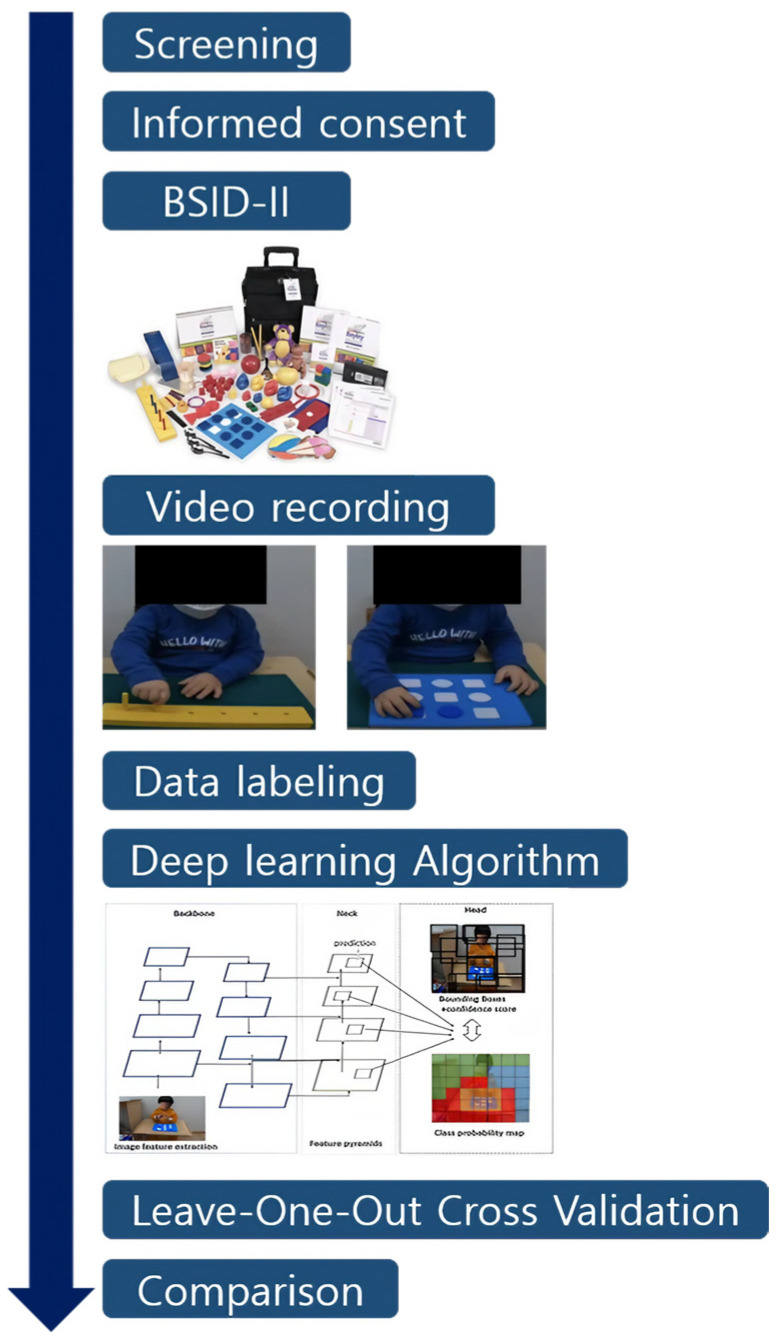
Study timeline.

This study was a single-center prospective cohort study conducted between July 2022 and December 2022. It was a pilot study, and children between the ages of 12 and 42 months who were referred to the outpatient clinic of Pediatric Rehabilitation Medicine for suspected DD were enrolled. Children with medical conditions that made it difficult for them to undergo video analysis and perform tasks were excluded. Written, informed consent was obtained by all the parents before the assessment, and the study was approved by the Institutional Review Board of Asan Medical Center (2022-0202). This research adhered to the study protocol, followed the Declaration of Helsinki, and complied with all relevant regulatory prerequisites. This study is registered with The Clinical Research Information Service (registration number: KCT0007614, date of first registration: 5 August 2022). Written informed consent for publication and participation was obtained from parents or legal guardians. Children were excluded from the study if they or their legal representative withdrew their consent to participate in the clinical trial. The timeline from screening to attendance to data analysis is illustrated in Figure 1.

### 2.2. Clinical Information

Clinical information of the children enrolled in this study was obtained, including gestational age, birth weight, sex, BSID-II test results, and participant age at the time of video analysis.

### 2.3. BSID

The BSID-II assessment was administered to all the enrolled children by an occupational therapist experienced in the diagnosis of DD. The assessment comprises two main subscales: the Mental Development Index (MDI) and the Psychomotor Development Index (PDI) [9,11,25]. The MDI assesses cognitive abilities such as memory, problem-solving, and language development. It includes tasks such as object permanence, imitation, and vocalization. The PDI assesses motor skills such as gross motor skills (e.g., crawling, walking) and fine motor skills (e.g., grasping, drawing). It includes tasks such as reaching for objects, stacking blocks, and drawing with a crayon. Scores on the MDI and PDI are reported as standardized scores, with a mean of 100 and a standard deviation of 15. The MDI of the BSID-II was used in this study to evaluate DD, and children were classified as normal (typically developing, MDI ≥ 85), having mild DD (70 ≤ MDI ≤ 84), and having significant DD (MDI ≤ 69) according to the BSID-II guidelines [9,11].

### 2.4. Video Recording of the Performances

**Figure 2:**
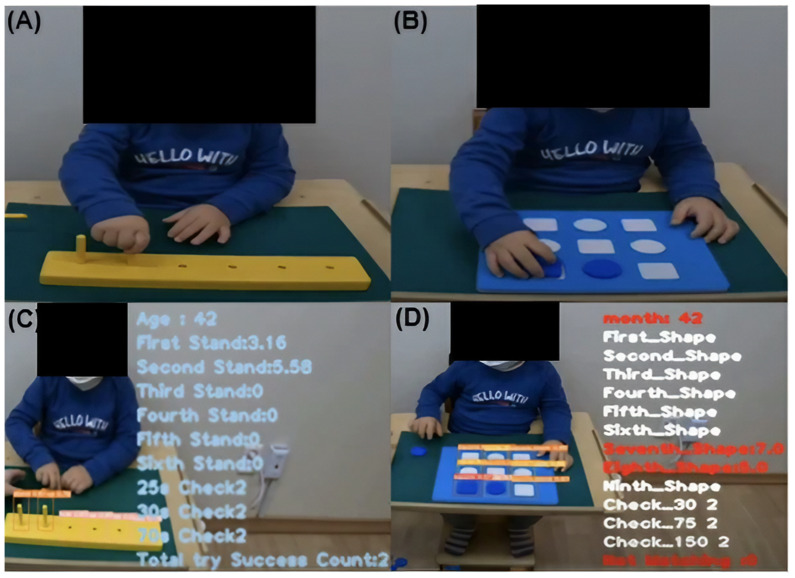
(**A**) Task “Place pegs in”. (**B**) Task “Blue Board”. (**C**) An algorithmic representation of performing Task (**A**) “Place pegs in”. (**D**) An algorithmic representation of performing Task (**B**) “Blue Board”.

Two tasks, which were part of the assessment using the BSID-II, were video recorded for analysis. In task (A), “Places Pegs in” (Figure 2A), the examiner handed the pegs to the child one by one, and each child was invited to move the 6 pegs to the empty spaces in the board. In the conventional BSID-II, children between 12 and 16 months old receive the credit (which means successful) when they place one or more pegs within 70 s (item 87). Children between 17 and 25 months old receive the credit when they place all six pegs within 70 s (item 98), and children over 26 months old receive the credit when they place all six pegs within 25 s (item 119).

In task (B), “Blue Board” (Figure 2B), the examiner handed puzzle pieces to the child and observed whether the child correctly placed 4 round and 5 square pieces in their respective shapes. In BSID-II, children between 14 and 19 months old receive the credit when they place one or more pieces within 150 s (item 90), children between 20 and 25 months old receive the credit when they place four or more pieces within 150 s, children between 26 and 28 months old receive the credit when they place nine pieces within 75 s (item 130), and children over 38 months old receive the credit when they place nine pieces within 30 s (item 165).

### 2.5. Camera Arrangement, Algorithm, Data Labeling, and Calibration

**Figure 3:**
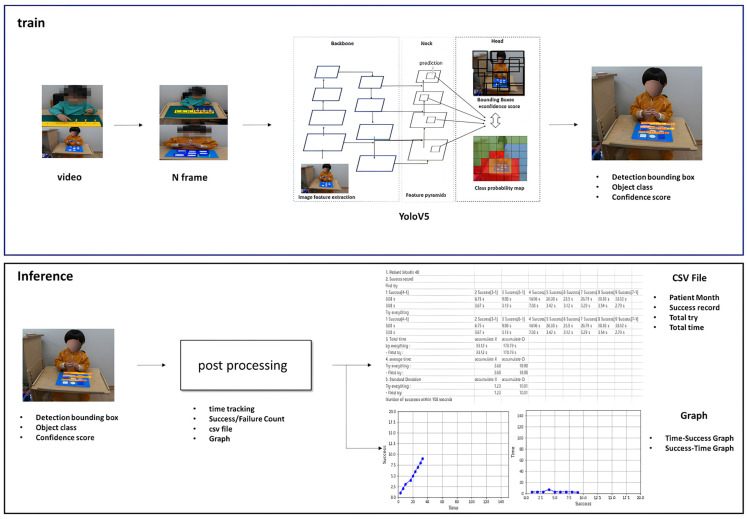
Video analysis algorithm overview.

The overall process, from video analysis to the comprehensive algorithmic presentation and data processing, is illustrated in Figure 3.

### 2.6. Data Smart Labeling

The labels required for this study were different for the “Places Pegs in” model and the “Blue Board” model. For the “Places Pegs in” model, we needed to specify and label the bounding box corresponding to the position of each ‘bar’, ‘hole’, and ‘stand’. In contrast, for the “Blue Board” model, we needed to specify and label the bounding box corresponding to the position of each ‘square’ and ‘circle’ on the panel, and each label was categorized as empty, well-shaped, or poorly-shaped. In this study, we used a smart labeling method that performed labeling on the videos for the first few cases, used these cases to train the model, obtained detection results for the remaining cases, and modified them again to train the model. The label dataset obtained was used to train the “Places Pegs in” and the “Blue Board” AI models.

### 2.7. YOLOv5 Model

YOLOv5 was deemed suitable for our project due to its improved performance, fast processing speed, user-friendly PyTorch 2.0-based structure, and the availability of multiple versions (S, M, L, XL) that can be adapted to different environments. In particular, YOLOv5 processes images at once, which allows it to consider the connectivity between consecutive frames rather than detecting objects in each frame of a video independently. For these reasons, our research team chose the YOLOv5 model. Figure 3 shows the overall structure of the YOLOv5 model. The model is composed of a Backbone, Neck, and Head section. In particular, this structure specializes in “bar model and stand” detection and shows high performance in effectively identifying the location of bar models and stands from different angles and situations.

### 2.8. “Places Pegs in” Algorithm

The algorithm starts by detecting the six holes in the bar and arranging them in ascending order from left to right. If a stand is detected instead of a hole, the time for that particular hole is recorded. After setting the location of the hole, the algorithm determines if the stand is within the hole by checking whether the center point of the stand falls within the area of the hole or not. If the stand is removed and replaced again, the time is reset to zero. The number of times the stand is inserted within 25, 30, and 70 s is recorded, as well as the total number of attempts to insert the stand.

### 2.9. “Blue Board” Algorithm

The algorithm starts by detecting the nine empty spaces on the board, storing the location and shape information (square or round) of each hole, and sorting them in ascending order from the top left. If a shape is detected at the location of a hole, the timing for that hole is recorded. The detected shape is represented by a bounding box (in the form of a rectangle), and a determination is made about whether the center point of this box lies inside the corresponding hole.

If the information in the shape matches the information in the hole, the timing is recorded for that shape. If the shape does not match the hole, the attempt is recorded as a “missed shape”. After removing the shape, the timing is reset if the location is detected as a hole again.

The number of shapes detected and matched during the set timings of 30, 75, and 150 s is recorded, as well as the total number of attempts to match shapes. In addition, the information on the number of attempts to insert shapes that do not match the hole is also recorded. In this way, the algorithm performs detection and analysis in real time and stores the necessary information.

### 2.10. Achievable Measurements Through Video Analysis

A. Parameters

In (A) “Places Pegs in” task, we measured the total number of attempts and time taken, as well as the number of holes the pegs went into and the time taken for each attempt. We recorded the maximum number of successful peg placements observed at 25, 35, and 75 s (Max). The success or failure of the tasks according to age in BSID-II items was recorded. In (B) “Blue Board” task, the maximum number of successful blocks observed at single time points (Max) were recorded at 30, 75, and 150 s, corresponding to Max(30), Max(75), and Max(150), respectively. In addition, the success or failure of the tasks according to age in BSID-II items was recorded. Fourteen children aged 12 months were not included in the sensitivity/specificity analysis due to the lack of applicable BSID-II items.

B. Real-time performance according to the age groups

We grouped children with normal results in the age-based MDI (MDI ≥ 85) into one group (TD group) and those with results indicating mild or significant delay (MDI ≤ 84) into another group (DD group). In addition, we divided the children into five age groups (12–17, 18–23, 24–29, 30–35, and 36–42 months) considering differences in performance by age. To reflect the child’s real-time performance of the task, we illustrated the number of successful peg insertions over time. Furthermore, for the “Places Pegs in” task, the accumulated distances between each hole were plotted over time according to the order in which the child inserted the pegs.

### 2.11. Statistical Methods

The baseline characteristics were presented using descriptive statistics. A 2 × 2 table indicates whether the child achieved success in (A) “Places Pegs in” and (B) “Blue Board” according to the conventional BSID-II and AI diagnosis. Based on this, the sensitivity, specificity, positive predictive value, and negative predictive value were calculated for each measurement [26]. In addition, subgroup analysis was performed by dividing children under 30 months into TD and DD groups according to MDI. Mean ± standard deviation and p-values were presented for each group using the Mann–Whitney test. All statistical calculations were conducted using SPSS (version 25, IBM Co., Armonk, NY, USA), and a *p*-value < 0.05 was considered statistically significant.

## 3. Results

### 3.1. Baseline Characteristics

**Figure 4:**
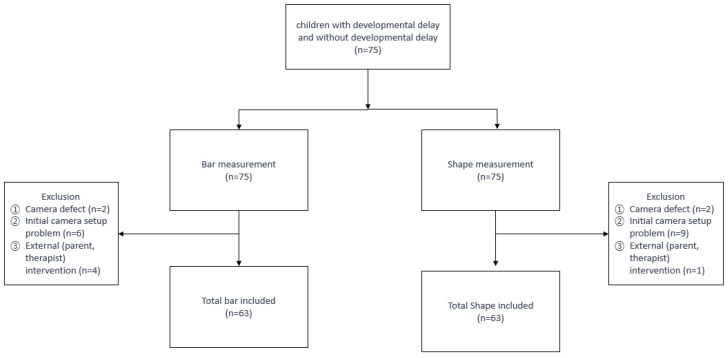
Flowchart.

A total of 75 children (43 male and 32 female) participated in the study with a mean gestational age at birth of 33 + 4 weeks, birth weight of 2355 ± 1027 g, and age at the time of video analysis of 25.2 ± 10.3 months (Table 1a) The mean values of MDI and PDI for the enrolled children were 88.9 ± 18.7 and 80.0 ± 16.7, respectively. The classification of subjects by age and MDI of BSID-II is presented in Table 1b. The flowchart and reasons for dropout for each test are described in Figure 4.

### 3.2. Places Pegs in

#### 3.2.1. Comparison of Accuracy Between the AI-Based Novel Tool and the Conventional BSID

As described in Table 2, out of 63 children, 37 children succeeded in task (A), “Places Pegs in”, of the BSID-II administered by the therapists. Of these, AI recorded 32 cases as successes and 5 as failures. Among the 26 children who did not meet the (A) “Places Pegs in” BSID-II criteria according to their age, AI recorded 26 cases as failures and 0 as successes. Therefore, using the data from the 2 × 2 table, the sensitivity was 86.5%, specificity was 100%, positive predictive value was 100%, negative predictive value was 83.9%, and accuracy was 92.1%. In the present study, Cronbach’s alpha of the “Places Pegs in” task was 0.91, and the Area Under the Curve (AUC) was 0.919 (Supplementary Figure S8).

#### 3.2.2. Real-Time Performance According to the Age Groups

**Figure 5:**
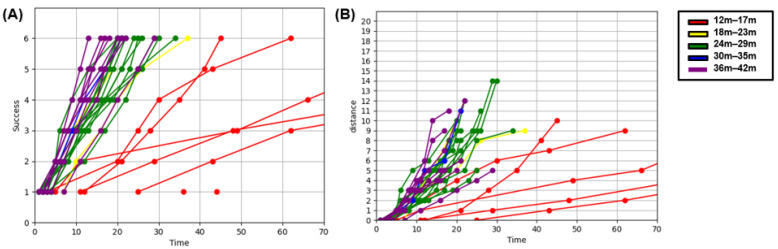
“Places Pegs in” in typically developing group. (**A**) Cumulative success X Time graph. (**B**) Cumulative distance X Time graph.

We divided the children with normal MDI (MDI ≥ 85) into five groups based on their age at enrollment and plotted the cumulative success over time on a graph (Figure 5A). The results showed differences in the slope (success/time) of each age group. Overall, the slope of the graph tended to become steeper as the age group increased. For children aged 30 months and older, there was a tendency for the graphs to overlap, indicating a lack of discriminatory power for the (A) task, which was considered an easy task for them. Additionally, we graphically represented the cumulative distance between successful pegs over time (Figure 5B).

### 3.3. Subgroup Analysis of Children Under 30 Months

We divided children under 30 months old into two groups (TD and DD groups) and compared performance, MDI, and PDI. In task (A), “Places Pegs in”, there were significant differences between the two groups in the maximum number of successful attempts at 25, 35, and 70 s, as well as in the MDI and PDI (Table 3).

### 3.4. Blue Board

#### 3.4.1. Comparison of Accuracy Between the AI-Based Novel Tool and the Conventional BSID

Table 2 represents the results of (B), “Blue Board” task, where out of 51 children, excluding those aged 12 months old, 32 children succeeded in the shape-matching task administered by the therapists. Of these, success was recorded for 31 children by AI, and failure was recorded for 1 child. Among the 19 children who did not pass the criteria for their BSID-II age group, AI recorded 17 cases as failures and 2 as successes. Accordingly, using the data from the 2 × 2 table, the sensitivity was 96.9%, specificity was 89.5%, positive predictive value was 94.0%, negative predictive value was 94.4%, and accuracy was 94.1%. In the present study, Cronbach’s alpha of the “Blue Board” task was 0.92, and the Area Under the Curve (AUC) was 0.929 (Supplementary Figure S8).

#### 3.4.2. Real-Time Performance According to the Age Groups

**Figure 6:**
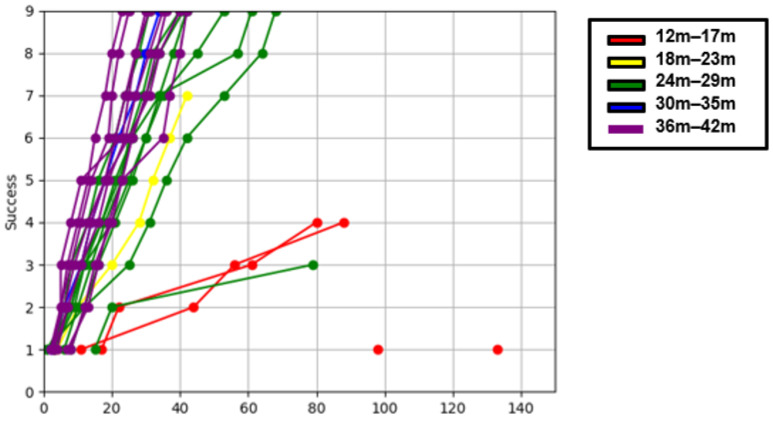
“Blue Board” cumulative success × time graph in typically developing group.

Using the same approach as that in task (A), “Places Pegs in”, we divided the children with normal MDI into five age groups and plotted the number of cumulative successes over time on a graph (Figure 6). We observed differences in the slope (success/time) based on age. In task (B), “Blue Board”, the amount of available data for children under 23 months old was small because of their limited participation in the task. Additionally, we further categorized the children into TD and DD groups based on MDI and graphically represented their performance (Supplementary Figure S2). The results revealed distinct differences in the slope (success/time) between the two groups in the age ranges of 24–29 months and 36–42 months.

#### 3.4.3. Subgroup Analysis of Children Under 30 Months

We divided children under 30 months old into two groups (TD and DD groups) and compared performance, MDI, and PDI. In task (B), “Blue Board”, significant differences were observed between the two groups in the MDI and PDI, but not in the maximum number of success cases at 30, 75, and 150 s. (Table 4).

## 4. Discussion

This prospective study demonstrated the feasibility of a novel AI-based video analysis of “Places Pegs in” and “Blue Board” items in the BSID-II in children aged 12–42 months. The AI-based tool showed a significant level of agreement with the traditional BSID-II (sensitivity of 86.5%, specificity of 100% for task (A), “Places Pegs in”, and a sensitivity of 96.9% and specificity of 89.5% for task (B), “Blue Board”. Furthermore, this novel tool suggested additional clinical information not available with the BSID-II, including real-time performance and automatic calculation of the maximum number of success cases observed at individual time points.

The high concordance observed between the novel tool and the existing BSID-II suggests that the novel tool can be useful in a clinical setting. The BSID, developed in 1969, is a well-established and validated developmental assessment tool [9,10,11,12]. However, it requires skilled individuals to administer and relies on the examiner’s interpretation of a child’s performance, leading to interrater variability [9,13,25,27]. This suggests that, particularly where the standard BSID is not feasible (for example, in remote areas with limited access to trained assessors), the unique ability of AI to analyze these BSID subtests could help assess development in children [9,12]. If the AI-based novel tool can be expanded to demonstrate high concordance with the BSID-II for items other than the (A) “Places Pegs in” and (B) “Blue Board” tasks, it has the potential to become a more objective and accurate assessment tool.

Previous studies have shown the potential of AI-based tools to assess the gross motor skills of children [19,20,21]. However, to the best of our knowledge, this study is the first to assess children’s development in fine motor and cognitive domains using an AI-based evaluation. In addition, this study demonstrated that the novel tool helped evaluate the performance of items in BSID-II. This result shows the potential to evolve into a more objective, sensitive, and accurate assessment tool for children. AI has the potential to revolutionize healthcare by improving diagnostic accuracy, speed, and accessibility, ultimately leading to better patient outcomes. Through a large cohort study, it will be possible to obtain data on children with normal development, and based on this, classification of developmental stages will be possible. In addition, new tools enable a more in-depth and comprehensive evaluation of child development, allowing accurate developmental assessment even in locations where experts are absent.

Furthermore, this AI-based novel tool developed in this study provided additional information compared to that assessed by the BSID. We were able to assess the real-time performance of children through the difference in slope on a graph (Figure 5 and Figure 6, Supplementary Figures S1 and S2). The slope becomes steeper as age increases. In this study, 63 children were analyzed and plotted, but it is difficult to draw clear boundaries due to the small number of patients. Rosenbaum et al. presented a gross motor function classification system for children with cerebral palsy using the GMFM-66 score [28]. There is still a long way to go, but if more sophisticated models are developed with more children to represent the distribution of real-time performance, it is expected that classification according to developmental level will be possible.

Additionally, we could observe the difference in performance between the TD and DD groups using tasks (A) “Places Pegs in” and (B) “Blue Board” through these analyses. This tool could also automatically calculate the maximum number of successes observed at certain time points; this aspect supports the view that the new assessment tool provides additional data compared to the conventional BSID-II. If sufficient data are collected using this innovative tool, it may help differentiate children with DD from the typically developing children.

Several limitations need to be acknowledged and considered in this study. Firstly, due to the small sample size, it was impossible to ensure an adequate data distribution by age group. Second, only two items of the BSID-II are included in this AI-based video analysis. These items were chosen because they reflect some of the cognitive, problem-solving, and fine motor skills. In addition, these two tasks are included in the item set for a wide range of ages (12 mo–42 mo) and are straightforward to analyze behavior using video at the tabletop. Nevertheless, the novel tool we proposed in this study does not fully capture the comprehensive nature of the data obtained from the BSID. Therefore, it is necessary to expand the items and domains of this AI-based video analysis in future studies to obtain more comprehensive data. Third, the two tasks chosen were relatively simple for children over 30 months of age, making it challenging to differentiate between the TD and DD groups. Further research is required to address these issues. Fourth, due to the characteristics of the recording environment, a different angle or distance of the camera set could result in low detection rates when using the trained AI model. This problem can be overcome by incorporating various training data or utilizing image augmentation techniques that can simulate camera angles and distances. Fifthly, the developmental assessment was carried out at a single point in time and was not repeated or followed up longitudinally. Finally, children who were unable to participate in this study due to unstable medical conditions or medical devices should be evaluated in future studies.

## 5. Conclusions

In this pilot study, a deep learning model showed high predictive accuracy for 2 items of the BSID, suggesting potential avenues for using deep learning-based software for objective developmental assessment. The application of AI has the potential to revolutionize healthcare by improving diagnostic accuracy, speed, and accessibility, which in turn could lead to better patient outcomes. Through a large cohort study, it will be possible to obtain data on typically developing children, and based on this, classification of developmental stages could be possible. If these techniques can be developed in an advanced and detailed form in the future, they will provide a deeper and more comprehensive assessment of a child’s development, allowing accurate developmental assessments to be made in places where experts are not available.

## Figures and Tables

**Table 1:** (**a**) Baseline characteristics of the study population (*n* = 75). (**b**) Classification of the subjects by age and MDI of BSID-II.

(**a**)				
**Variables**	**Mean ± SD**			
Gestational age				
<28 weeks	14 (18.7)			
28 ≤ GA < 32 weeks	11 (14.7)			
32 ≤ GA < 37 weeks	23 (30.7)			
>37 weeks	27 (36.0)			
Birth weight (g)	2355 ± 1027			
Age (at the time of participation, months)	25.2 ± 10.3			
Sex (Male: Female)	43 (57.3): 32 (43.7)			
Mental developmental index	88.9 ± 18.7			
Psychomotor developmental index	80.0 ± 16.7			
(**b**)				
	**MDI of BSID**			
**Age**	**Typically Developing**	**Mild Delay**	**Significant Delay**	**Total**
12–17 months	15	7	3	25
18–23 months	1	3	2	6
24–29 months	11	2	3	16
30–35 months	2	3	3	8
36–42 months	8	5	7	20
Total	37	20	18	75

Abbreviation: BSID, Bayley Scales of Infant Development; MDI, Mental Developmental Index. Values are mean ± SD or number (%).

**Table 2:** Comparison of accuracy between BSID-II and deep learning.

Methods	Results, No				Validation Measure, %				
	True Positive	False Positive	True Negative	False Negative	Sensitivity	Specificity	PPV	NPV	Accuracy
“Places Pegs in” (*n* = 63)	32	0	26	5	86.5	100	100	83.9	92.1
“Blue Board” (*n* = 51)	31	2	17	1	96.9	89.5	94.0	94.4	94.1

Abbreviation: PPV: positive predictive value; NPV: negative predictive value.

**Table 3:** Subgroup analysis of children under 30 months in “Places Pegs in” task.

	TD Group (*N* = 25)	DD Group (*N* = 17)	
Variables	Mean ± SD	Mean ± SD	*p*-Value
MDI	98.44 ± 10.10	72.20 ± 14.58	0.00 *
PDI	87.68 ± 14.85	71.94 ± 18.92	0.01 *
Max (25)	2.64 ± 2.68	1.12 ± 2.09	0.04 *
Max (35)	3.00 ± 2.81	1.35 ± 2.21	0.04 *
Max (70)	3.52 ± 2.97	1.82 ± 2.48	0.04 *

Values are mean ± SD. * *p* < 0.05 by Mann–Whitney test. Abbreviation: TD: Typically Developing; DD: Developmental Delay; MDI: Mental developmental index; Psychomotor developmental index; Max: maximum number of successful attempts.

**Table 4:** Subgroup analysis of children under 30 months in “Blue Board” task.

	TD Group (*N* = 20)	DD Group (*N* = 18)	
Variables	Mean ± SD	Mean ± SD	*p*-Value
MDI	100.00 ± 9.49	72.92 ± 14.75	0.00 *
PDI	89.05 ± 14.27	70.50 ± 18.43	0.00 *
Max (30)	1.85 ± 2.43	0.89 ± 1.57	0.15
Max (75)	3.30 ± 4.07	1.94 ± 3.39	0.28
Max (150)	3.63 ± 3.94	1.85 ± 3.36	0.25

Values are mean ± SD. * *p* < 0.05 by Mann–Whitney test. Abbreviation: TD: Typically Developing; DD: Developmental Delay; MDI: Mental developmental index; Psychomotor developmental index; Max: maximum number of successful attempts.

## Data Availability

The data supporting the conclusions of this study can be obtained from the corresponding author upon reasonable request due to privacy.

## Supplementary Materials

The following supporting information can be downloaded at: https://www.mdpi.com/article/10.3390/children12030276/s1, Figure S1: Comparing performance over time between the normal and DD groups in the ‘Places Peg In’ task; Figure S2: Comparing performance over time between the normal and delay groups in the “Blue Board” task; Figure S3: Gopro Camera arrangement; Figure S4: bar PR curve; Figure S5: shape PR curve; Figure S6: “Places pegs in” confusion matrix; Figure S7: “Blue Board” confusion matrix; Figure S8: Receiver Operating Characteristic (ROC) curve and Area Under the Curve (AUC) of two tasks, [29,30,31].
