# Supplementary material for: Can AI-Based Video Analysis Help Evaluate the Performance of the Items in the Bayley Scales of Infant Development?
Source: Children (Basel). 2025 Feb 25;12(3):276. doi: 10.3390/children12030276 (PMC11941028; doi:10.3390/children12030276)
Supplement: Supplementary file 1 [file children-12-00276-s001.zip › children-3442538-supplementary.pdf]

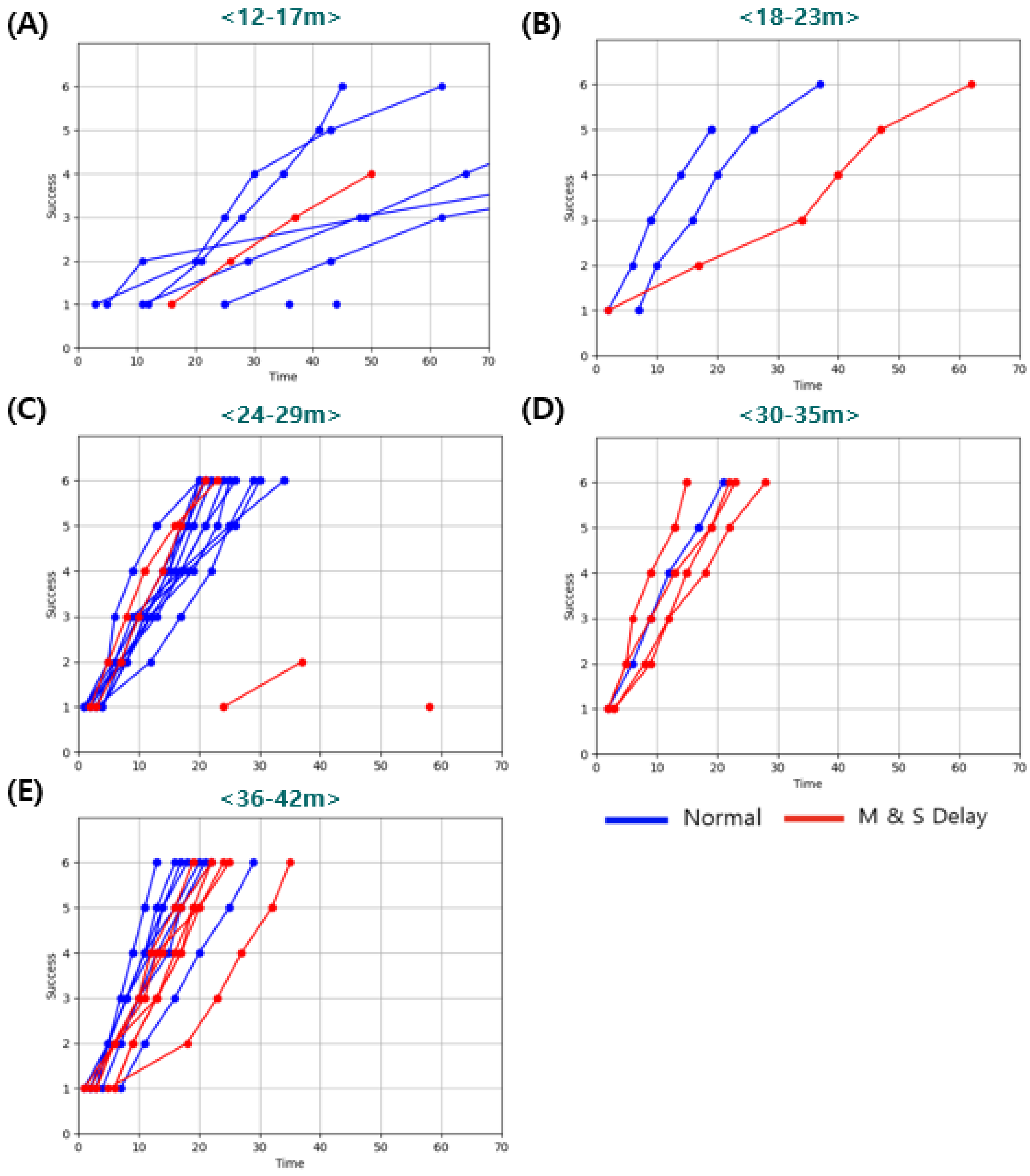

Supplementary figure S1. Comparing performance over time between the normal and DD groups in the 'Places Peg In' task: (A) 12-17 months, (B) 18-23 months, (C) 24-29 months, (D) 30-35 months, and (E) 36-42 months

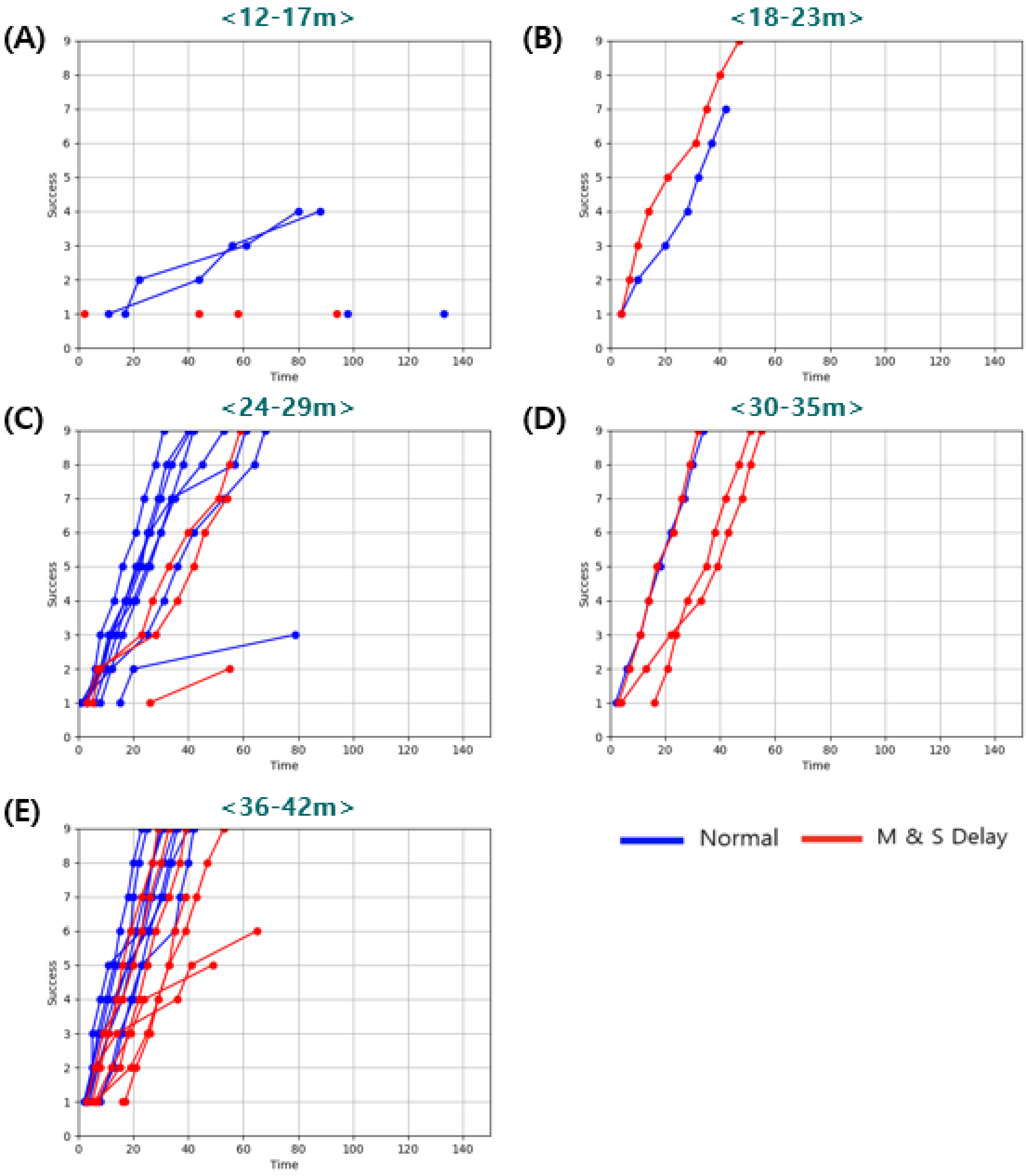

Supplementary figure S2. Comparing performance over time between the normal and delay groups in the "Blue Board" task: (A) 12-17 months, (B) 18-23 months, (C) 24-29 months, (D) 30-35 months, and (E) 36-42 months

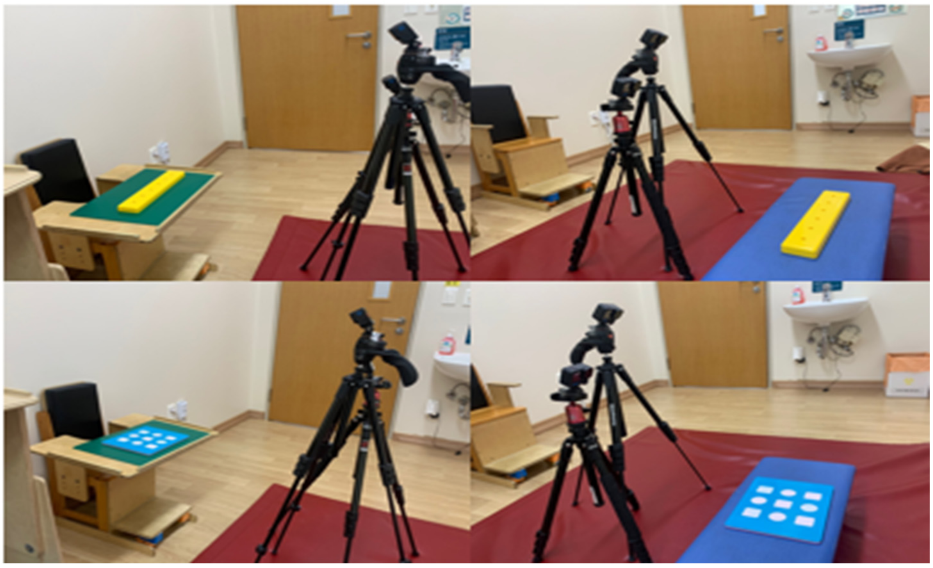

Supplementary figure S3. Gopro Camera arrangement.

YOLOv5 model

(1) YOLOv5 applies various scaling and transformations in the data preprocessing stage to improve detection performance for small objects. In particular, when training the model, we standardize the learning process by resizing the input images to a uniform dimension. This initial dimension is typically set to 512 x 512 x 3, which allows for the effective detection of objects of different sizes and shapes.

(2) The core structure of the Backbone network consists of a Focus structure and a Cross Stage Partial (CSP) structure[1, 2]. The Focus structure slices the image before it enters the Backbone network. For example, if the original image is 512 x 512 x 3, it is sliced to produce a feature map of 256 x 256 x 12, which is then subjected to a convolution operation. The Focus operation can downsample the input dimensions without parameters while retaining as much information as possible from the original image. The CSP structure uses two 1x1 convolutions for the transformation of the feature map. This structure improves the learning ability of convolutional networks while helping to eliminate computational bottlenecks and reduce memory costs.

(3) The Neck section is the network layer that combines the features of the image and passes them to the prediction layer. In YOLO v5, the Neck consists of a Feature Pyramid Network (FPN) and a Path Aggregation Network (PAN) structure[3]. The FPN up-samples high-level feature information and fuses it with feature maps at different scales in a top-to-bottom fashion. This produces feature maps at different scales for prediction. PAN uses a bottom-to-top approach to pass strong positional information from lower layers to higher layers[3]. This is helpful in determining the exact location of an object. This combination of FPN and PAN helps to detect and localize objects of different sizes and shapes more accurately.

(4) The head plays a key role in the final stage of object detection, where the actual object detection and classification are done based on the features extracted from the backbone and neck. First, it estimates Bounding Boxes. These boxes represent the location and size of the detected objects, and each Bounding Box is output with a Confidence Score. Next, we generate a Class Probability Map for each Bounding Box. Finally, the Head section generates information about the location of the object (Bounding Boxes), the probability that the object exists at that location (Confidence Score), and the type of object (Class Probability Map), all of which are interpreted together to perform the final object detection and classification.

(5) The camera placement for "Places Pegs in" includes two GoPro cameras placed slightly above the patient, facing left and right **(Supplementary figure S3)**. The cameras are placed on a tripod and adjusted in height using a height-adjustable tripod to ensure that the patient has a clear view of the holes in the pegs and stand.

Experiment

The performance requirements of deep learning are typically very high. In this experiment, we used an NVIDIA TITAN RTX card, which is one of NVIDIA's high-performance cards. We chose the NVIDIA card because of its excellent framework support. The CUDA version used was 11.0, and the PyTorch version used was 1.7.1+cu110. The processor used was a 13th Gen Intel Core i5-13600KF with a clock speed of 3.50 GHz. The operating system was 64-bit Windows 11 Pro, with a x64-based processor. The total RAM was 32.0 GB (31.8 GB available). Python version 3.8.16 was used as the programming language.

We applied the Leave-One-Out Cross Validation (LOOCV) method to train and evaluate the performance of the model. The LOOCV method is known to be particularly useful in situations where data are sparse.

Using the frames for each patient, we used Intersection over Union (IOU) as a metric to compare each model in LOOCV with the final trained model. We set the threshold for IOU to a commonly used value of 0.5, which measures the degree of overlap between two bounding boxes, and only if it is above 0.5 was the bounding box correctly detected.

The IOU-based accuracy between the final model and the LOOCV model was found to be around 99%, which means that the two models were performing quite similarly. Therefore, we further computed other performance metrics such as Mean Average Precision (mAP) and Confusion Matrix for LOOCV to conduct a broader performance evaluation.

In this study, we selected precision and mean average precision (mAP) as the main evaluation metrics. Precision is an important metric for accurately evaluating an algorithm's ability to localize and detect targets.

$$\text{mAP} = \frac{1}{N}\sum_{i=1}^{n} AP_i$$

where i represents each class, n is the number of total classes, and AP_i represents the average precision for the i-th class.

In a PR curve, P represents precision and R represents recall. This curve is a visual representation of the relationship between an algorithm's precision and recall. Typically, recall is on the x-axis (horizontal axis) and precision is on the y-axis (vertical axis).

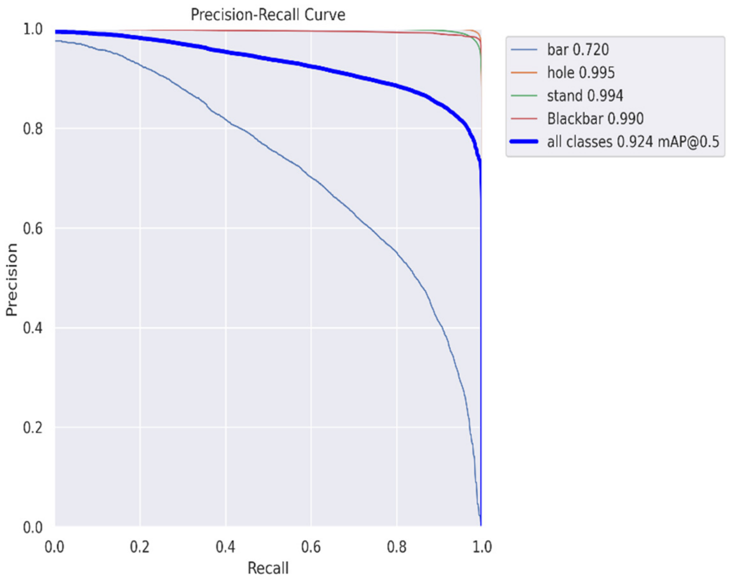

Supplementary figure S4. bar PR curve

Supplementary figure S4 shows the Precision-Recall Curve used in the 'bar' study. We saw high numbers in the evaluations for 'hole', 'stand', and 'Blackbar'. In particular, for 'bar', the corner of the bar was detected when the child was holding and moving the bar. In the early stages of the study, we detected this area to track the movement of the bar, but we did not include it in the final evaluation metric.

Represents the Precision-Recall Curve used in the 'shape' study. We see high values for 'square', 'circle', 'square blank', 'circle_blank', and 'Black_Bar'.

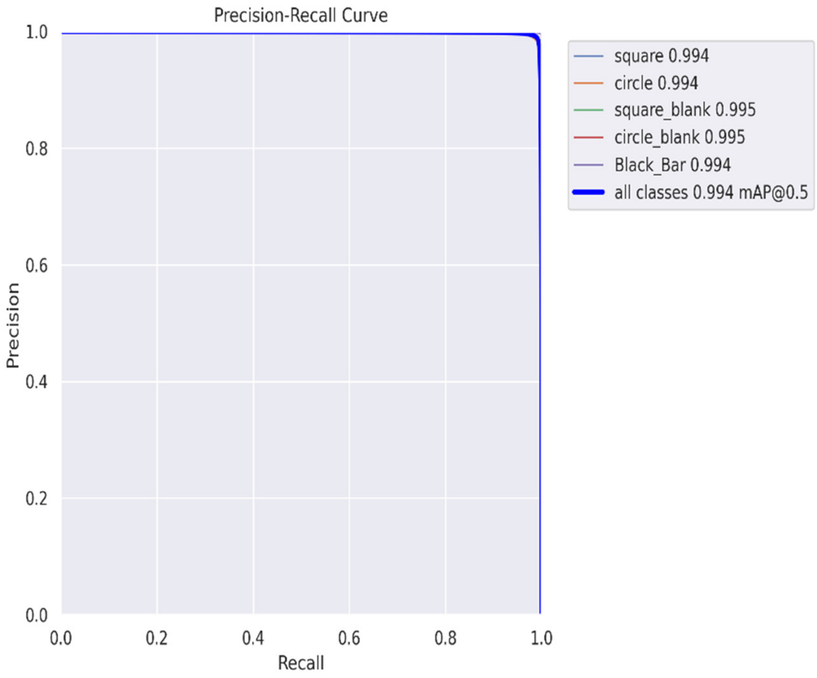

Supplementary figure S5. shape PR curve

The confusion matrix is one of the main tools for evaluating the performance of a classification algorithm. This matrix clearly shows how well the algorithm classifies and what types of errors it tends to make. The main components are True Positive, True Negative, False Positive, and False Negative. From these values, we can derive various performance metrics such as precision, recall, and accuracy of the classification algorithm. In particular, in this study, we plotted the confusion matrix for the 'bar' model and the 'shape' model, and found that both models performed well.

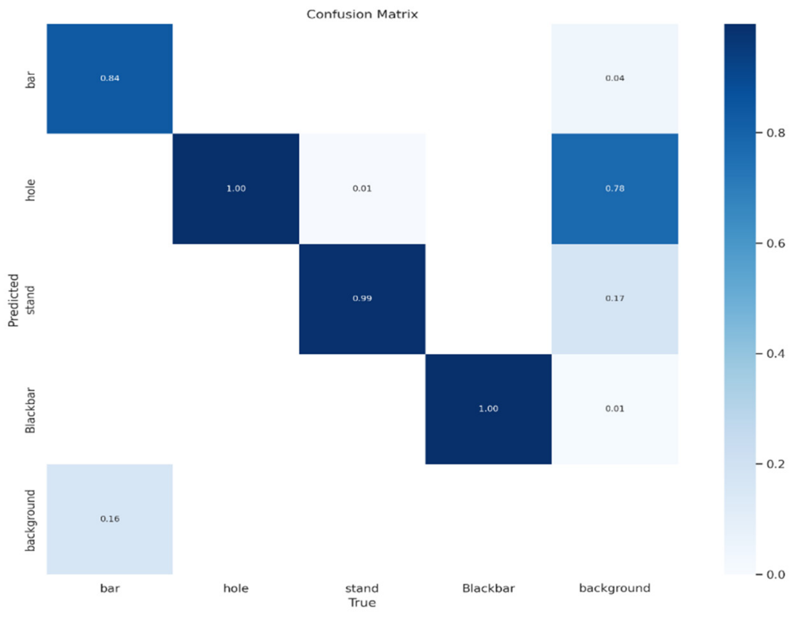

Supplementary figure S6. "Places pegs in" confusion matrix

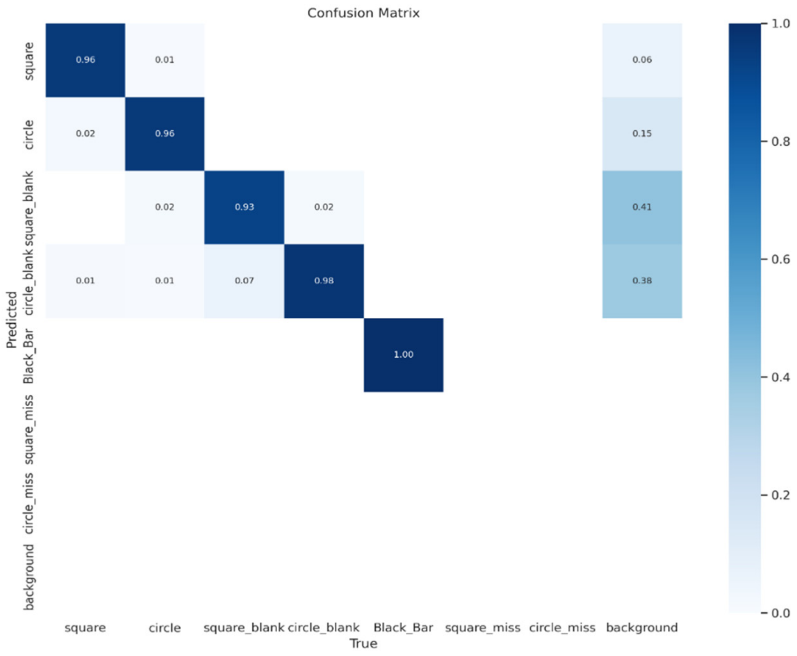

Supplementary figure S7. "Blue Board" confusion matrix

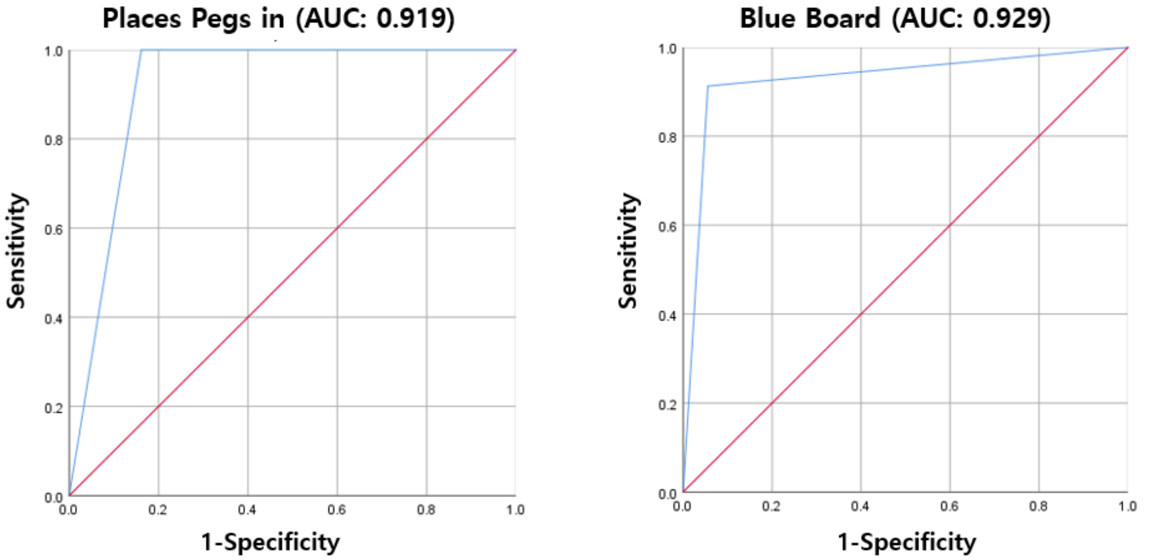

Supplementary figure S8.

Receiver Operating Characteristic (ROC) curve and Area Under the Curve (AUC) of two tasks

1. Yang, S.J., et al., *Assessing microscope image focus quality with deep learning.* BMC bioinformatics, 2018. **19**: p. 1-9.
2. Wang, C.-Y., et al. *CSPNet: A new backbone that can enhance learning capability of CNN*. in *Proceedings of the IEEE/CVF conference on computer vision and pattern recognition workshops*. 2020.
3. Wang, K., et al. *Panet: Few-shot image semantic segmentation with prototype alignment*. in *proceedings of the IEEE/CVF international conference on computer vision*. 2019.
